# Phenotypic aging mediates the association between neutrophil percentage-to-albumin ratio and muscular dystrophies: a population-based study

**DOI:** 10.3389/fneur.2025.1599600

**Published:** 2025-10-21

**Authors:** Hongyang Gong, Jiajun Wang, Xiaomei Lin, Shaoqun Huang

**Affiliations:** ^1^Department of Oncology Surgery, Fuzhou Hospital of Traditional Chinese Medicine Affiliated to Fujian University of Traditional Chinese Medicine, Fuzhou, Fujian, China; ^2^Department of Physiology, College of Medicine, Chosun University, Gwangju, Republic of Korea; ^3^First Clinical Medical College, Fujian University of Traditional Chinese Medicine, Fuzhou, Fujian, China; ^4^Department of Orthopedics, Fuzhou Hospital of Traditional Chinese Medicine Affiliated to Fujian University of Traditional Chinese Medicine, Fuzhou, Fujian, China

**Keywords:** muscular dystrophies, neutrophil percentage-to-albumin ratio, phenotypic age, NHANES, mediation analysis

## Abstract

**Background:**

Current research on Muscular Dystrophies (MD) suggests that its occurrence is closely related to several mechanisms, including gene mutations, dystrophin deficiency, and alterations in the Akt/mTOR/p70S6K signaling pathway. The development of MD is influenced by factors such as genetics, inflammation, age, and underlying conditions. However, few studies have explored the association between the Neutrophil Percentage-to-Albumin Ratio (NPAR), phenotypic age, and MD. This study investigates the relationship between NPAR and MD and evaluates whether phenotypic age influences this association.

**Methods:**

Subgroup analysis, restricted cubic splines (RCS), and multivariable logistic regression were employed to explore the relationship between NPAR and MD. Additionally, mediation analysis was conducted to investigate the potential role of phenotypic age in the relationship between NPAR and MD.

**Results:**

A total of 3,416 participants were included in this study, among whom 268 cases of MD were reported (6%, weighted). After adjusting for all variables using multivariable logistic regression, each unit increase in NPAR and phenotypic age was associated with a 29% (OR = 1.29, 95% CI: 1.19–1.39) and 7% (OR = 1.07, 95% CI: 1.03–1.12) increase in the odds of muscular dystrophies, respectively. Consistent results were obtained when NPAR and phenotypic age were categorized into tertiles, with a *p* for trend < 0.001. Restricted cubic spline (RCS) analysis indicated a linear positive correlation between NPAR and MD prevalence. Mediation analysis showed that 22.26% of the relationship between NPAR and MD was mediated by phenotypic age (*p* = 0.026).

**Conclusion:**

This study found a significant positive correlation between NPAR and MD, with phenotypic age acting as a partial mediator in this relationship. This finding underscores the potential of NPAR as a predictive marker for MD prevalence and highlights the importance of promoting healthy aging to reduce the risk of MD.

## 1 Introduction

Muscular dystrophies (MD) refer to a group of heterogeneous genetic disorders characterized by similar clinical and dystrophic pathological features observed in muscle biopsies (1). These conditions are commonly associated with age-related loss of skeletal muscle mass, strength, and function (2). While MD shares a common pathology of muscular dystrophy, it can be divided into different subtypes based on varying clinical symptoms and genetic defects. The current classification of MD is primarily based on clinical presentation and age of onset (3). Epidemiological studies have found that the most frequent form in childhood is Duchenne muscular dystrophy (DMD), with an incidence rate of approximately 8.92 per 100,000 people. In adults, the most common type is myotonic dystrophy, with an incidence rate of about 10.6 per 100,000 people (3). The characteristic clinical manifestation of MD is progressive muscle degeneration, which may affect not only limb skeletal muscles but also respiratory muscles, cardiac smooth muscles, and swallowing muscles in certain subtypes. In some cases, MD can also involve the brain, inner ear, eyes, or skin, leading to a range of complications (3). Therefore, early diagnosis of MD and identification of related risk factors are crucial for treatment and improving the quality of life for affected individuals.

The Neutrophil Percentage-to-Albumin Ratio (NPAR) is a blood biochemical marker that reflects the ratio of neutrophils to albumin, serving as an indicator of inflammation, infection, and nutritional status in patients. As a novel marker for both inflammation and nutritional assessment, NPAR has a dual function in reflecting inflammatory cytokines and nutritional markers. Zhou and his colleagues’ study on inflammatory markers for predicting mortality in chronic obstructive pulmonary disease (COPD) demonstrated that both NPAR and NLR (neutrophil-to-lymphocyte ratio) could predict mortality, with NPAR showing superior predictive performance (4). Similarly, a study by Wang and his colleagues found that NPAR could predict mortality in patients at risk of acute kidney injury (AKI) (5). NPAR has also been used as an inflammation-based prognostic indicator in patients with cardiogenic shock and those undergoing palliative care for pancreatic cancer (6). Furthermore, some studies have found that NPAR may reflect the onset and prognosis of depression (7). In summary, NPAR is a novel and convenient diagnostic tool with broad clinical applications, warranting further research to confirm its general applicability and accuracy across various diseases.

Phenotypic Age is calculated by integrating chronological age with nine hematological biomarkers: glucose, albumin, creatinine, alkaline phosphatase, C-reactive protein, red cell distribution width, mean corpuscular volume, white blood cell count, and lymphocyte percentage (8). Given the overlap in components, NPAR may share a similar relationship with Phenotypic Age. Additionally, studies have shown that muscle mass and function decline with age (9). Therefore, it is hypothesized that Phenotypic Age is positively correlated with the onset and progression of MD. As NPAR reflects both systemic inflammation and nutritional status, proving its association with MD could be of great significance for developing diagnostic and therapeutic monitoring tools for MD. This study systematically analyzes data from the 2011–2018 National Health and Nutrition Examination Survey (NHANES) to uncover the deeper relationship between NPAR and MD. The findings aim to provide a reference for future diagnostic, therapeutic, and prevention strategies for MD.

## 2 Methods

### 2.1 Study participants

The National Health and Nutrition Examination Survey (NHANES) is a continuous, stratified, multistage sampling program designed to assess the health and nutritional status of adults and children in the United States, covering a wide range of health and nutritional indicators. Each year, NHANES conducts a nationally representative sampling survey involving approximately 5,000 individuals, primarily consisting of interviews and physical examination components. The interview portion addresses demographic, socioeconomic, dietary, and health-related issues, while the physical examination includes physiological measurements and laboratory tests. All participants are required to provide written informed consent. The NHANES research project is reviewed and approved by the Research Ethics Review Board of the National Center for Health Statistics to ensure scientific integrity and ethical compliance.

Among the 39,156 participants across the four NHANES cycles from 2011 to 2018, individuals under 20 years of age and pregnant women were excluded (*n* = 16,539). Further exclusions were made for those lacking NPAR data (n = 2,395), Phenotypic Age data (*n* = 15,552), and MD data (*n* = 1,254). Ultimately, a total of 3,416 participants were included in the final analysis (Supplementary Figure S1).

### 2.2 Definition of NPAR

NPAR is defined as the logarithmic ratio of the percentage of neutrophils in plasma to the albumin level, calculated using the following formula: NPAR = Percentage of Neutrophils (of total white blood cell count) × 100/Albumin (g/dL) (7). A Coulter® HMX was used to do a full blood count (CBC). The CBC profile in the NHANES database was used to determine hematological parameters, such as albumin and neutrophil count.

### 2.3 Definition of phenotypic age

According to previous studies (8), Phenotypic Age is a composite metric based on multiple biomarkers (laboratory examination indicators) used to assess an individual’s biological age. The calculation process involves nine clinical biomarkers, including white blood cell count, mean corpuscular volume, red blood cell distribution width, alkaline phosphatase, triglycerides, C-reactive protein, glucose, albumin, and creatinine, along with chronological age.

The calculation formula is as follows:


$$\text{Phenotypic}\;\mathrm{Age}=141.50+\frac{\ln[-0.00553\times \ln(1-\mathrm{xb})]}{0.09165}$$

Where: xb = −19.907–0.0336 × albumin+0.0095 × creatinine +0.0195 × glucose+0.0954 × ln(CRP) − 0.0120 × lymphocyte percent.

+0.0268 × mean cell volume+0.3356 × red blood cell distribution width+0.00188 × alkaline phosphatase+0.0554 × white blood cell count+0.0804 × chronological age.

### 2.4 MD assessment

The primary outcome of this study was the presence of muscular dystrophies (MD). NHANES itself does not provide a direct diagnostic variable for MD. Instead, we followed a recently published NHANES-based study (doi: 10.3389/fnut.2024.1465486) that applied previously established criteria. Briefly, whole-body dual-energy X-ray absorptiometry (DEXA) examination files were used to calculate appendicular lean mass (ALM). ALM refers to the muscle weight of the limbs (arms and legs), typically measured using dual-energy X-ray absorptiometry (DXA), with data available from NHANES examination results. The ALM/BMI ratio is widely recognized as an effective indicator for assessing muscle condition. According to this metric, the diagnostic criteria for muscular dystrophy are defined as follows (2, 10): an ALM/BMI ratio below 0.789 for males and below 0.512 for females. This approach not only considers muscle mass but also accounts for differences in body composition, providing a more comprehensive and accurate assessment of muscle status.

### 2.5 Covariates

In this study, the covariates included were age, sex, race, marital status, education level, poverty income ratio, smoking status, physical activity, hypertension, diabetes, and hyperlipidemia. For further details, please refer to Supplementary Table S1.

### 2.6 Statistical analysis

Statistical analyses in this study were conducted using R (version 4.3.1), with all analyses employing sampling weights to ensure that the estimated data is nationally representative. The weight variable fasting subsample 2-year weight (WTSAF2YR) was utilized, with the new weight for the years 2011–2018 calculated as 1/4 × WTSAF2YR (11). Continuous variables are presented as mean ± standard deviation, and *p*-values were calculated using t-tests. Percentages and p-values for categorical variables (weighted N, %) were determined using weighted chi-square tests. Multivariable logistic regression models were constructed to analyze the relationships between NPAR and MD, as well as between Phenotypic Age and MD. Three models were established: (1) a crude model without covariate adjustment; (2) a model adjusted for age, sex, education level, marital status, PIR, and race; and (3) a model further adjusted for smoking status, physical activity, hypertension, diabetes, and hyperlipidemia. Smoothing spline fitting was employed to explore the linear or nonlinear relationship between NPAR and MD. Subgroup analyses were conducted to investigate the relationship between NPAR and MD. Mediation analysis was performed to evaluate the indirect, direct, and overall effects of Phenotypic Age in mediating the relationship between NPAR and MD, with the mediation proportion calculated as indirect effect/ (indirect effect + direct effect) × 100%. The mediation effects were computed using the “mediation” package in R (11). A two-tailed *p*-value of less than 0.05 was considered statistically significant.

## 3 Result

### 3.1 Baseline characteristics

This study included 3,416 participants aged 20 years and older, which extrapolates to approximately 41.83 million adults in the United States. The prevalence of MD was found to be 6%, representing about 2.54 million individuals. Statistically significant differences (*p* < 0.05) were observed among MD patients in terms of race, education level, income level, hypertension, diabetes, and hyperlipidemia. Additionally, the NPAR levels and Phenotypic Age were higher in the MD group compared to the non-MD group. Further details can be found in Tables 1, 2. The unweighted baseline characteristics are presented in Supplementary Table S2.

**Table 1:** Baseline characteristics of all participants were stratified by muscular dystrophies, describing the participants’ socio-demographic characteristics (weighted).

Characteristic	Overall, *N* = 41,836,397 (100%)	Non-Muscular dystrophies, *N* = 39,288,758 (94%)	Muscular dystrophies, *N* = 2,547,639 (6%)	*p* value
No. of participants in the sample	3,416	3,148	268	–
Age (%)				0.245
20–30	16,430,237 (39)	15,556,216 (40)	874,021 (34)	
31–40	13,463,909 (32)	12,651,534 (32)	812,375 (32)	
>40	11,942,251 (29)	11,081,008 (28)	861,243 (34)	
Gender (%)				0.113
Male	20,914,427 (50)	19,468,601 (50)	1,445,825 (57)	
Female	20,921,971 (50)	19,820,157 (50)	1,101,814 (43)	
Race (%)				<0.001
Other	8,087,459 (19)	7,374,712 (19)	712,748 (28)	
Non-Hispanic White	24,025,656 (57)	23,126,518 (59)	899,138 (35)	
Non-Hispanic Black	4,591,694 (11)	4,535,929 (12)	55,765 (2)	
Mexican American	5,131,588 (13)	4,251,599 (10)	879,988 (35)	
Married/live with partner (%)				0.500
No	16,349,039 (39)	15,413,865 (39)	935,175 (37)	
Yes	25,487,358 (61)	23,874,894 (61)	1,612,464 (63)	
Education level (%)				<0.001
Below high school	4,893,171 (12)	4,186,919 (11)	706,253 (28)	
High school or above	36,943,226 (88)	35,101,840 (89)	1,841,386 (72)	
PIR (%)				0.002
Not Poor	29,917,123 (77)	28,417,271 (78)	1,499,852 (66)	
Poor	8,781,305 (23)	8,012,744 (22)	768,561 (34)	

Mean (SD) for continuous variables: the *p* value was calculated by the weighted Students *t*-test. Percentages (weighted *N*, %) for categorical variables: the p value was calculated by the weighted chi-square test. PIR, Ratio of family income to poverty.

**Table 2:** Baseline characteristics of all participants were stratified by muscular dystrophies, describing lifestyle habits, co-morbidities, and NPAR and phenotypic age (weighted).

Characteristic	Overall, *N* = 41,836,397 (100%)	Non-Muscular dystrophies, *N* = 39,288,758 (94%)	Muscular dystrophies, *N* = 2,547,639 (6%)	*p* value
No. of participants in the sample	3,416	3,148	268	–
Smoking (%)				0.976
Never	25,649,029 (62)	24,066,892 (61)	1,582,137 (62)	
Former	7,631,421 (18)	7,178,934 (18)	452,487 (18)	
Current	8,555,947 (20)	8,042,932 (21)	513,015 (20)	
Physical activity (%)				0.056
Inactive	3,722,563 (11)	3,438,550 (10)	284,014 (15)	
Active	31,588,508 (89)	30,035,410 (90)	1,553,098 (85)	
Hypertension (%)				0.001
No	33,899,335 (81)	32,124,002 (82)	1,775,334 (70)	
Yes	7,937,062 (19)	7,164,757 (18)	772,306 (30)	
Diabetes (%)				<0.001
No	39,487,840 (94)	37,291,701 (95)	2,196,139 (86)	
Yes	2,348,558 (6)	1,997,057 (5)	351,501 (14)	
Hyperlipidemia (%)				<0.001
no	19,486,655 (47)	18,751,596 (48)	735,059 (29)	
yes	22,349,742 (53)	20,537,162 (52)	1,812,580 (71)	
Neutrophil percentage [mean (SD)]	57.18 (8.62)	57.04 (8.65)	59.27 (7.84)	<0.001
Albumin_(g/dL) [mean (SD)]	4.32 (0.35)	4.32 (0.35)	4.18 (0.37)	<0.001
NPAR [mean (SD)]	13.34 (2.30)	13.27 (2.29)	14.30 (2.35)	<0.001
NPAR (%)				<0.001
T1	13,912,939 (34)	13,393,956 (34)	518,982 (21)	
T2	13,993,788 (33)	13,250,474 (34)	743,314 (29)	
T3	13,929,671 (33)	12,644,328 (32)	1,285,343 (50)	
Phenotypic age [mean (SD)]	32.11 (9.80)	31.92 (9.77)	34.97 (9.81)	<0.001
Phenotypic age (%)				0.001
T1	13,948,614 (33)	13,394,702 (34)	553,912 (22)	
T2	13,957,249 (33)	13,010,872 (33)	946,378 (37)	
T3	13,930,535 (34)	12,883,185 (33)	1,047,350 (41 s)	

Mean (SD) for continuous variables: the *p* value was calculated by the weighted Students *t*-test. Percentages (weighted *N*, %) for categorical variables: the *p* value was calculated by the weighted chi-square test. NPAR, neutrophil percentage-to-albumin ratio.

### 3.2 Association between NPAR, phenotypic age, and MD

As shown in Table 3, three different models were employed to evaluate the association between NPAR and MD, all indicating a positive correlation between NPAR and the prevalence of MD (all *p* < 0.001). In Model 3, after adjusting for various covariates, each one-unit increase in NPAR was associated with 29% higher odds of MD [OR: 1.29 (95% CI: 1.19–1.39)].

**Table 3:** Association between NPAR, phenotypic age, and muscular dystrophies, NHANES 2011–2018.

Characteristics	Model 1 [OR (95% CI)]	*p*-value	Model 2 [OR (95% CI)]	*p*-value	Model 3 [OR (95% CI)]	*p*-value
NPAR - Muscular dystrophies						
Continuous	1.21 (1.11–1.31)	<0.001	1.23 (1.11–1.36)	<0.001	1.29 (1.19–1.39)	<0.001
Tertile						
T1	1 (ref.)		1 (ref.)		1 (ref.)	
T2	1.45 (0.85–2.46)	0.170	1.43 (0.75–2.74)	0.260	1.82 (0.88–3.74)	0.100
T3	2.62 (1.59,4.33)	<0.001	2.74 (1.38–5.47)	0.010	3.62 (1.77–7.41)	0.002
*p* for trend	<0.001		0.004		<0.001	
Phenotypic age - Muscular dystrophies						
Continuous	1.03 (1.02–1.05)	<0.001	1.07 (1.03–1.12)	0.002	1.07 (1.03–1.12)	0.003
Tertile						
T1	1 (ref.)		1 (ref.)		1 (ref.)	
T2	1.76 (1.20–2.57)	0.010	2.29 (1.29–4.08)	0.010	2.25 (1.14–4.44)	0.020
T3	1.97 (1.33–2.90)	0.001	3.14 (1.44–6.87)	0.010	3.58 (1.41–9.11)	0.010
*p* for trend	<0.001		0.010		0.010	

Model 1: no covariates were adjusted. Model 2: age, gender, education level, marital status, PIR, and race were adjusted. Model 3: age, gender, education level, marital status, PIR, race, smoking, physical activity, hypertension, diabetes, and hyperlipidemia were adjusted. NPAR, neutrophil percentage-to-albumin ratio; PIR, Ratio of family income to poverty; OR, odds ratio; CI, confidence interval.

Furthermore, when NPAR was categorized into tertiles, the group with the highest NPAR (T3) showed a 3.62-fold increase in MD odds compared to the group with the lowest NPAR (T1) [OR: 3.62 (95% CI: 1.77–7.41)].

**Figure 1:**
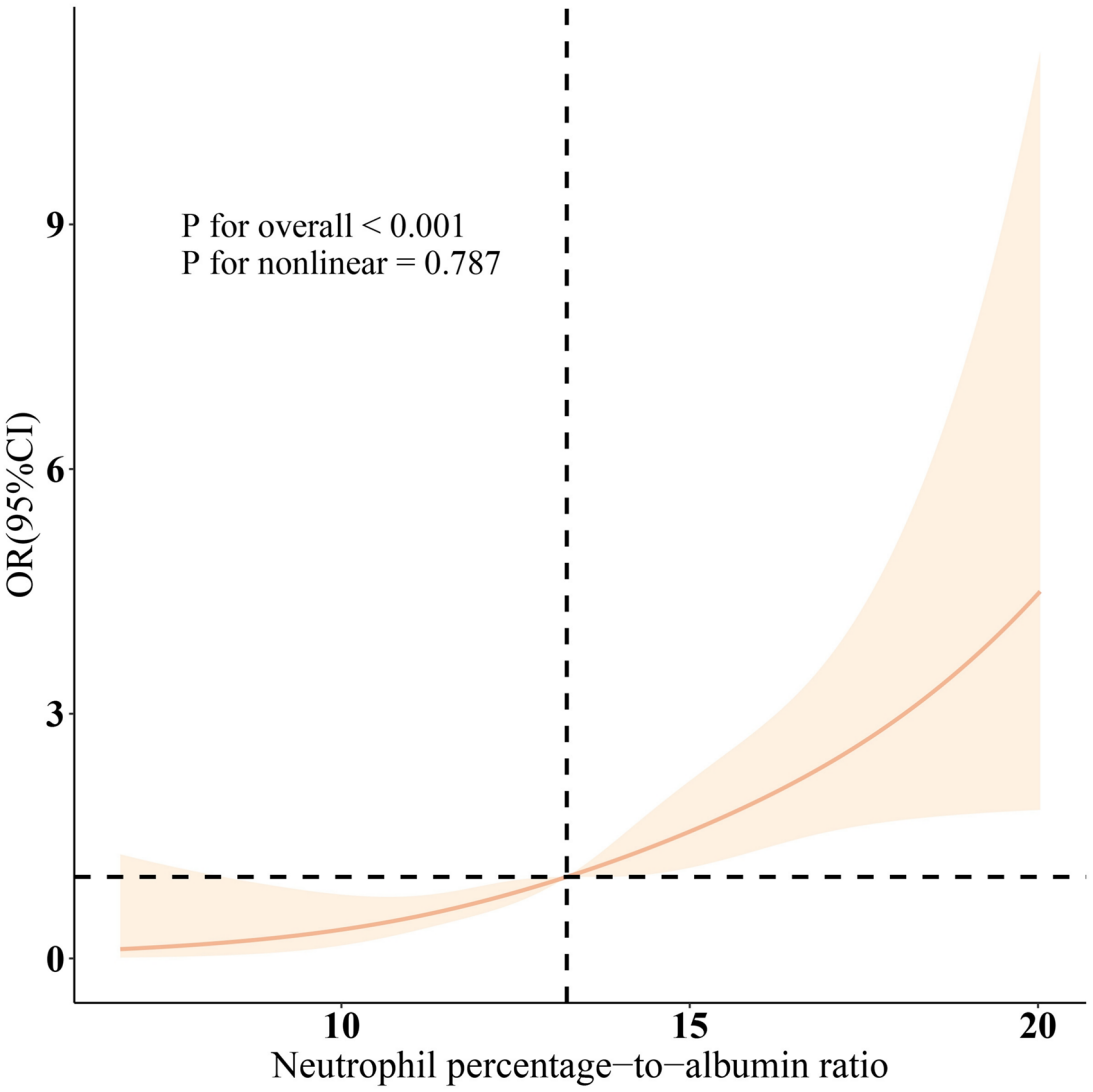
Dose–response relationships between NPAR and Muscular dystrophies. OR (solid lines) and 95% confidence levels (shaded areas) were adjusted for age, gender, education level, marital status, PIR, race, smoking, physical activity, hypertension, diabetes, and hyperlipidemia.

Additionally, the relationship between Phenotypic Age and MD was assessed, revealing a positive correlation across all three models (all *p* < 0.05). As Phenotypic Age increased, the prevalence of MD also increased, with statistically significant results (all p < 0.05). The results from the restricted cubic spline (RCS) analysis (Figure 1) further demonstrated a significant linear positive correlation between NPAR and the prevalence of MD after adjusting for relevant variables (overall *p* < 0.001; nonlinearity *p* = 0.787).

**Figure 2:**
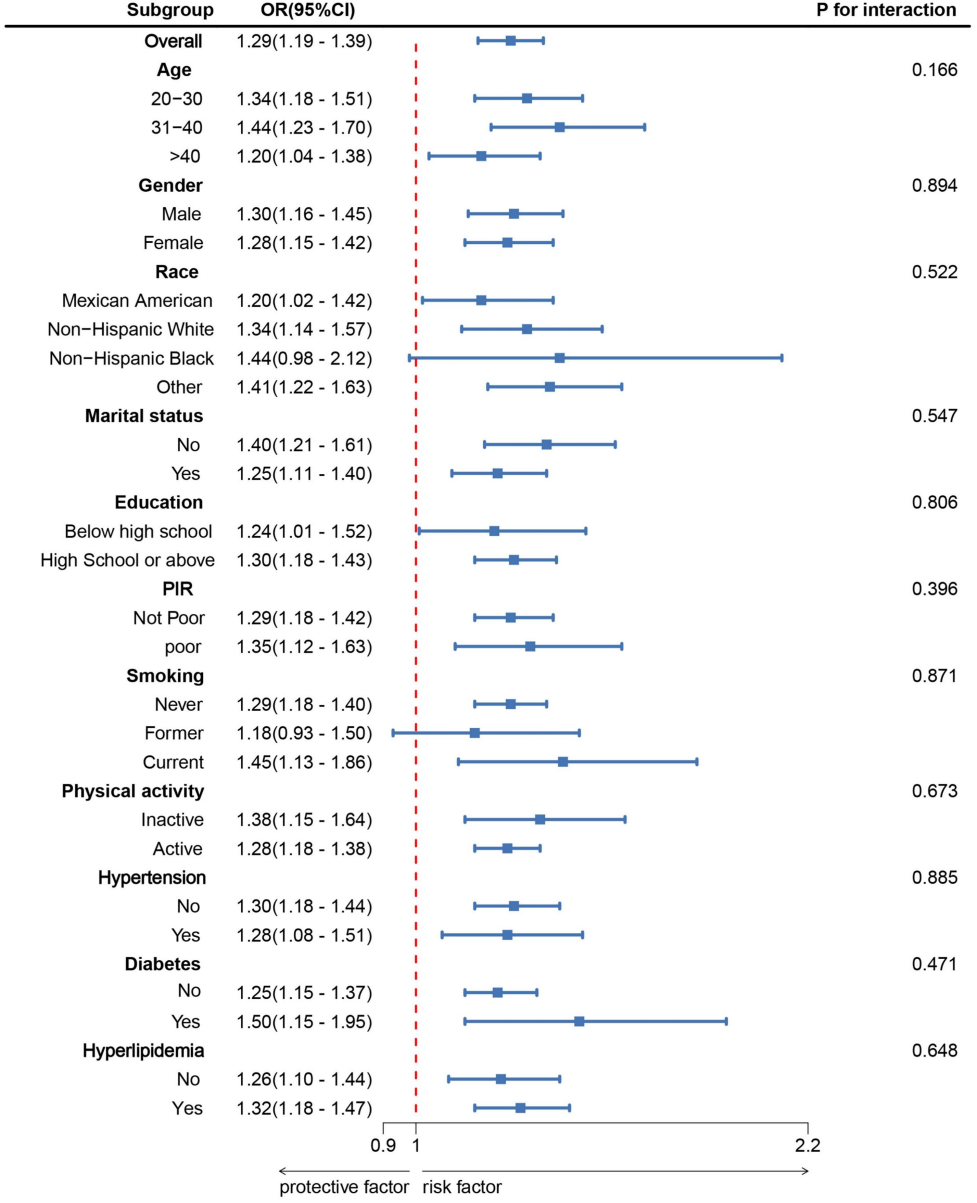
Subgroup analysis between NPAR and Muscular dystrophies. ORs were calculated for each 1 unit increase in NPAR. Analyses were adjusted for age, gender, education level, marital status, PIR, race, smoking, physical activity, hypertension, diabetes, and hyperlipidemia.

Subgroup analyses were conducted based on age, sex, race, marital status, education level, PIR, smoking status, physical activity, hypertension, diabetes, and hyperlipidemia (Figure 2). The results indicated a significant positive correlation between NPAR and MD across all subgroups. Moreover, no interactions were observed within the subgroups (*p* > 0.05).

### 3.3 Mediation effect

**Figure 3:**
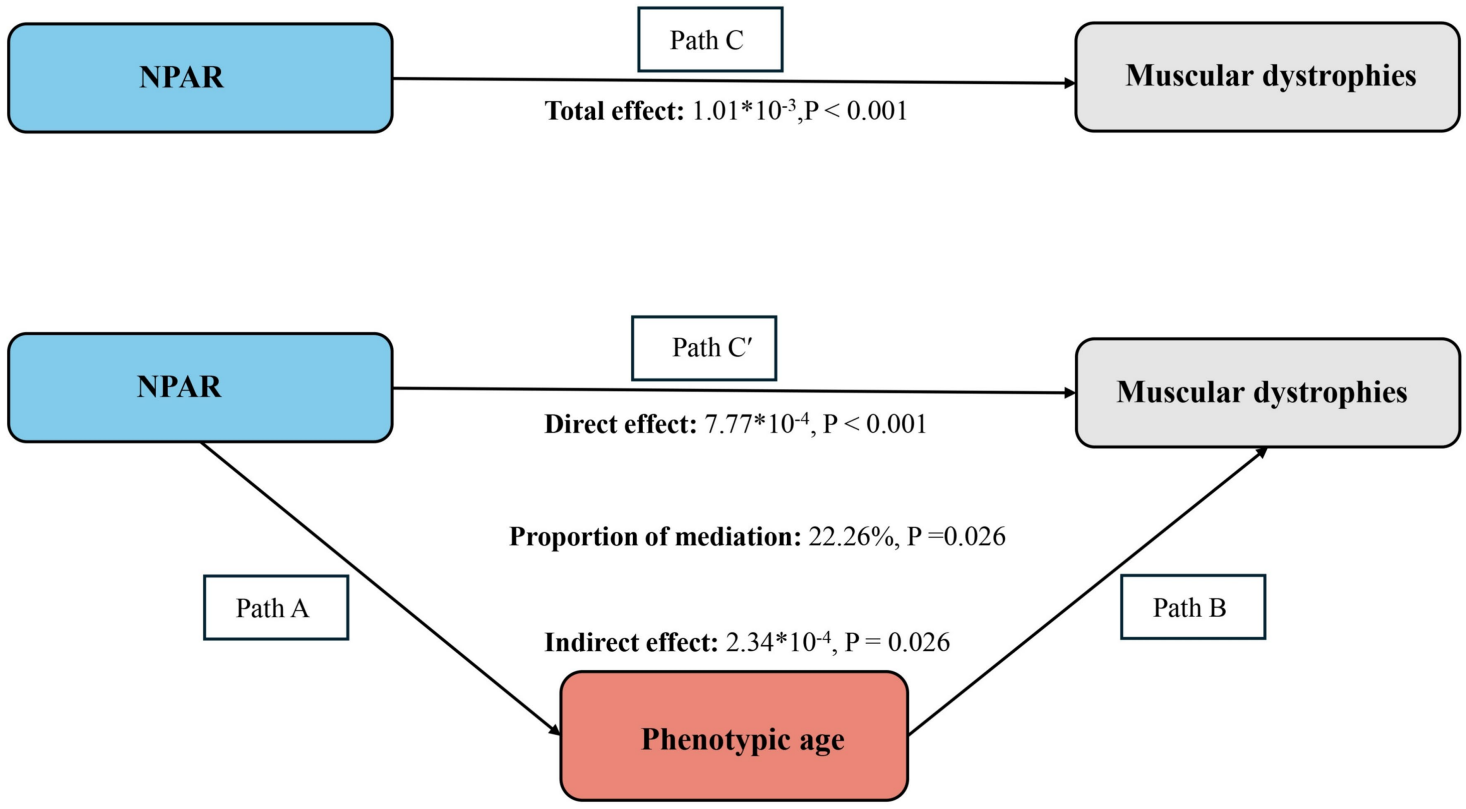
Schematic diagram of the mediation effect analysis. Path C indicates the total effect; path C’ indicates the direct effect. The indirect effect is estimated as the multiplication of paths A and B (path A*B). The mediated proportion is calculated as indirect effect/ (indirect effect + direct effect) × 100%. Abbreviation: NPAR, neutrophil percentage-to-albumin ratio. Analyses were adjusted for age, gender, education level, marital status, PIR, race, smoking, physical activity, hypertension, diabetes, and hyperlipidemia.

The mediation model is illustrated in Figure 3, where NPAR, MD, and Phenotypic Age serve as the independent variable, dependent variable, and mediator, respectively. Table 4 shows that, after adjusting for other covariates, there is a significant correlation between NPAR and Phenotypic Age (*β* = 1.20, 95% CI: 1.10–1.30).

**Table 4:** Multivariate linear regression of NPAR and Phenotypic age.

	*β*	95% CI	*p*-value
NPAR - Phenotypic age	1.20	(1.10-s1.30)	0.007

Adjusted for age, gender, education level, marital status, PIR, race, smoking, physical activity, hypertension, diabetes, and hyperlipidemia.

After adjusting for all covariates, the mediating effect of Phenotypic Age was observed (indirect effect = 2.34 × 10^−4^, *p* = 0.026; direct effect = 7.77 × 10^−4^, *p* < 0.001), resulting in a mediation proportion of 22.26% (*p* = 0.026). Thus, the Phenotypic Age can be considered a mediating factor in the association between NPAR and MD.

## 4 Discussion

In this representative study of U. S. adults, a significant positive correlation was found between NPAR, Phenotypic Age, and MD. Furthermore, mediation analysis indicated that Phenotypic Age serves as a mediating factor in the relationship between NPAR and MD, suggesting that Phenotypic Age may play a key role in the pathway through which NPAR influences MD.

Current research indicates that MD are primarily influenced by genetic factors. Notably, conditions such as DMD and certain forms of limb-girdle muscular dystrophy can present significant symptoms during childhood. The predominant mechanism underlying DMD is attributed to the absence or dysfunction of a protein known as dystrophin, with the gene segment encoding this protein referred to as the DMD locus. Subsequent studies have also identified variations in this locus as being associated with Becker muscular dystrophy (BMD) and several other genetic disorders (12–14). Furthermore, existing research has found that the higher prevalence of myotonic dystrophy among adults is related to deficiencies in protein kinase, leading to reduced phosphorylation of membrane proteins. Myotonic dystrophy is recognized as an autosomal dominant disorder, with its pathogenesis linked to the expansion of a CTG trinucleotide repeat sequence within the untranslated 3′ region of the DMPK gene, resulting in abnormal protein kinase production (15, 16).

At the protein factor pathway level, some studies suggest that alterations in the skeletal muscle Akt/mTOR/p70S6K signaling pathway activity may also contribute to the development of muscular dystrophies. Specifically, research has shown that reduced Akt protein activity in DMD model mice leads to diminished activity throughout this signaling pathway, resulting in impaired skeletal muscle synthesis. Analyses correlating the changes in the Akt/mTOR/p70S6K signaling pathway with the pathological alterations in skeletal muscle of myotonic dystrophy type 1 (DM1) patients have revealed extensive activation of this pathway, which consequently leads to pathological hypertrophy of muscle fibers (17, 18). Another pathway under investigation highlights that mutations in glycosylphosphatidylinositol-anchored protein B (GMPPB) can result in a muscular dystrophy variant characterized by low glycosylation of *α*-dystroglycan (α-DG). Guanosine diphosphate-mannose is essential for the mannosylation of proteins, including α-DG, and serves as a substrate for cytosolic mannosyltransferases (19).

Currently, there is a significant gap in research regarding the interconnections and mechanisms between NPAR and muscular dystrophies (MD). Neutrophils, as classic effector cells, play a crucial role in mediating inflammatory responses. Inflammation has been demonstrated to be closely associated with various cardiovascular diseases. NPAR, as an indicator reflecting cardiac function and myocardial injury, offers greater clinical sensitivity compared to simply focusing on the ratios of neutrophils and albumin levels (20). MD can manifest in multiple phenotypes that simultaneously lead to dysfunction of cardiac smooth muscle. For instance, Emery-Dreifuss muscular dystrophy (EDMD) typically presents with a triad of progressive muscle weakness, joint contractures, and cardiac abnormalities (21). Severe DMD can also result in dilated cardiomyopathy, further progressing to heart failure, while Becker muscular dystrophy is similarly associated with related cardiac pathologies (22). Additionally, myotonic dystrophy can induce cardiac damage, leading to ventricular dysfunction as well as supraventricular and ventricular arrhythmias (16, 23, 24). Therefore, NPAR theoretically has the potential to serve as an indicator for assessing the progression of muscular dystrophies.

Research has indicated a significant association between phenotypic age and the progression of MD. The reduction in muscle size and strength, diminished protein synthesis, and increased apoptosis rates in muscle cells are clear characteristics of aging observed in both human and animal models. Furthermore, studies have found that advancing age may affect molecular signaling pathways within the muscle, such as the dysfunction of the Akt/protein kinase B pathway, which can further influence muscle metabolism and function (25–27). These molecular mechanisms provide a new perspective on understanding the relationship between age and muscular dystrophy. As people age, muscle mass reduction is a common phenomenon. Research on the effects of aging on muscle structure indicates that elderly individuals experience muscle atrophy, characterized by lower muscle mass and cross-sectional area, alongside a decrease in both the number and size of muscle fibers (25). The impact of aging on muscle protein synthesis is significant. One major hypothesis regarding the progressive decline in muscle quality with age is that older muscles are more susceptible to injury compared to younger muscles, yet their recovery capacity declines (28). The aging process severely impairs skeletal muscle regeneration (29). Studies have shown that older animals exhibit slower regeneration rates after injury and generate lower quantities and functional quality of regenerated muscle compared to younger animals. Additionally, aging is accompanied by increased muscle catabolism and shifts in muscle fiber types (30, 31). Older adults also display diminished responses to amino acids and insulin stimuli, further impacting muscle protein synthesis (32, 33). Therefore, it can be concluded that the impact of aging on protein synthesis in MD may lead to significant exacerbation of the disease and its symptoms. This may explain one of the reasons for the observed correlation between phenotypic age and MD.

This study utilized data from the 2011–2018 NHANES, a nationally representative weighted sample, which enhances the applicability and generalizability of our findings. By incorporating phenotypic age as a mediating factor, we were able to conduct a more in-depth analysis of the relationship between NPAR and MD. Additionally, the exploration of underlying mechanisms and pathways has provided insights into the association between these two variables, potentially guiding future research into the pathological mechanisms linking NPAR and MD. We have also thoroughly considered and controlled for potential confounding factors, ensuring the robustness and reliability of our results. Currently, there are still limitations in clinical assessments and systematic treatment strategies for MD. Through this study, we aim to identify new clinical indicators for MD and contribute to the prevention of disease progression. In addition to the previously discussed link with cardiac smooth muscle, the association between NPAR and MD may also involve neutrophil-driven inflammation and albumin-related nutritional status. Neutrophils and their subtypes can influence muscle atrophy and regeneration through pro-inflammatory cytokines and oxidative stress, while low albumin may impair muscle protein synthesis. These factors likely contribute to the observed relationship between elevated NPAR and MD, warranting further investigation in future studies. Phenotypic age may capture aspects of muscle aging, including loss of muscle mass, reduced strength, and impaired regeneration. These age-related changes likely contribute to the development and progression of MD, supporting the role of PA as a mediator between systemic aging processes and skeletal muscle deterioration.

This study has several limitations. First, due to the cross-sectional design, this study cannot establish causality between NPAR, phenotypic age, and MD. Second, this study was unable to eliminate all potential confounding factors, such as lifestyle, dietary habits, and genetic factors, which may simultaneously influence all three variables. The NHANES database lacks detailed information on these aspects. Moreover, certain severe forms of MD, such as DMD and BMD, primarily manifest in childhood and can lead to significant developmental abnormalities or even mortality. However, most biochemical indicators for children are not available in the NHANES database, which posed challenges for this study. Consequently, we did not include children in our analysis, but we plan to focus on the pediatric population in future research. Additionally, NHANES only represents the U. S. population, and therefore, our findings may not be generalizable to populations in Europe, Asia, Africa, or other regions regarding the prevalence of MD. Another issue that should be acknowledged is the potential overlap between NPAR and phenotypic age (PA). Since NPAR incorporates serum albumin, and albumin is also a component of PA, part of the mediation effect we observed may be partially driven by this shared biomarker. This overlap could introduce ambiguity in mediator selection and may limit the ability to fully disentangle the independent role of PA as a mediator. Therefore, the mediation effect should be interpreted with caution. Future studies are warranted to validate these findings using alternative indices of systemic inflammation and nutritional status that do not directly overlap with PA components, as well as longitudinal data to further clarify causal mechanisms. It should be noted that the NHANES database does not provide direct clinical diagnostic data on muscular dystrophy. Therefore, the definition of muscular dystrophy in our analysis was based on previously published NHANES studies, which may not fully capture the clinical diagnostic criteria. This approach, although consistent with prior literature, could lead to potential misclassification and limit the generalizability of our findings. Additionally, because the definitions of sarcopenia and muscular dystrophy share overlapping features in terms of muscle weakness and functional decline, some degree of conceptual ambiguity may remain. Therefore, our findings should be interpreted with caution, and further investigations using clinically validated diagnostic criteria for muscular dystrophy are warranted to confirm and extend our results. Another limitation of our study is the presence of missing data in some covariates. While participants with missing information on the core variables (Phenotypic aging, NPAR, and muscular dystrophy) were excluded, those with missing values in other covariates were retained to preserve sample size and statistical power. Although this strategy likely had only a minor influence on the primary associations, it may still introduce potential bias. Future studies should consider applying multiple imputation methods to better address missing data.

## 5 Conclusion

In conclusion, this study found a strong positive correlation between NPAR and MD, highlighting the potential of NPAR as a predictive indicator for MD prevalence. Phenotypic age, as a measure of biological age, reflects an individual’s actual aging status, which is significant in today’s increasingly aging society. By introducing phenotypic age as a mediating factor, this study was able to further explore the mechanistic connections between NPAR and MD. These findings suggest that the NPAR-phenotypic age-MD relationship may serve as an important pathway for studying factors associated with MD.

## Data Availability

The datasets presented in this study can be found in online repositories. The names of the repository/repositories and accession number(s) can be found at: https://wwwn.cdc.gov/nchs/nhanes/.
