# Supplementary material for: Phenotypic aging mediates the association between neutrophil percentage-to-albumin ratio and muscular dystrophies: a population-based study
Source: Front Neurol. 2025 Oct 21;16:1599600. doi: 10.3389/fneur.2025.1599600 (PMC12583221; doi:10.3389/fneur.2025.1599600)
Supplement: Supplementary file 1 [file Data_Sheet_1.docx]

**Supplementary Material**

**Table S1.** Description of covariates.

| Covariates | Description in NHANES |
| --- | --- |
| Age | Divided into three groups: 20-30 years old, 31-40  years old, >40 years old |
| Gender | Male and Female |
| Race | Mexican American, Non-Hispanic Black, Non-Hispanic White, Other Race |
| Educational level | Below high school, High School or above |
| Marital status | Yes: Married/Living with partner; No: Never married/Separated/Divorced/Widowed |
| PIR | Poor: <1.3; Not Poor:>=1.3 |
| Smoking | Smoking status was grouped into never smoker (defined as <100 cigarettes in a lifetime), current smoker (defined as ≥100 cigarettes in a lifetime), and former smoker (defined as ≥100 cigarettes and had quit smoking) |
| Physical activity | Active physical activity was defined as >599 MET, or >149 min of moderate physical activity, or >74 min of vigorous physical activity |
| Diabetes | Diabetes was defined as a history of previous diabetes, HbA1c level ≥6.5%, or fasting blood glucose level ≥126 mg/dL |
| Hypertension | The diagnostic criteria consist of self-reported hypertension history, the utilization of antihypertensive medication, a systolic blood pressure (SBP) ≥ 140mmHg, or a diastolic blood pressure (DBP) ≥ 90mmHg |
| Hyperlipidemia | (1) Triglyceride (TG) levels ≥150 mg/dl (1.7 mmol/L);(2) Total cholesterol (TC) levels ≥200 mg/dl (5.18 mmol/L);(3) Low-density lipoprotein (LDL) levels ≥130 mg/dl (3.37 mmol/L);(4) High-density lipoprotein (HDL) levels: Men: <40 mg/dl (1.04 mmol/L); Women: <50 mg/dl (1.30 mmol/L);(5) Individuals taking cholesterol-lowering drugs are also considered hyperlipidemia. |

PIR, Ratio of family income to poverty.

**Table S2.** Baseline characteristics of all participants were stratified by Muscular dystrophies, Unweighted.

| **Characteristic** | **Overall**, N = 3416 (100%) | **Non-Muscular dystrophies**, N = 3148 (92.2%) | **Muscular dystrophies**, N = 268 (7.8%) | **P Value** |
| --- | --- | --- | --- | --- |
| **Age (%)** |  |  |  | **0.001** |
| *20-30* | 1,282 (38%) | 1,202 (38%) | 80 (30%) |  |
| *31-40* | 1,128 (33%) | 1,044 (33%) | 84 (31%) |  |
| *>40* | 1,006 (29%) | 902 (29%) | 104 (39%) |  |
| **Gender (%)** |  |  |  | 0.089 |
| *Male* | 1,640 (48%) | 1,498 (48%) | 142 (53%) |  |
| *Female* | 1,776 (52%) | 1,650 (52%) | 126 (47%) |  |
| **Race (%)** |  |  |  | **<0.001** |
| *Other* | 1,097 (32%) | 1,006 (32%) | 91 (34%) |  |
| *Non-Hispanic White* | 1,038 (30%) | 984 (31%) | 54 (20%) |  |
| *Non-Hispanic Black* | 671 (20%) | 661 (21%) | 10 (3.7%) |  |
| *Mexican American* | 610 (18%) | 497 (16%) | 113 (42%) |  |
| **Married/live with partner (%)** |  |  |  | **0.024** |
| *No* | 1,368 (40%) | 1,278 (41%) | 90 (34%) |  |
| *Yes* | 2,048 (60%) | 1,870 (59%) | 178 (66%) |  |
| **Education level (%)** |  |  |  | **<0.001** |
| *Below high school* | 593 (17%) | 499 (16%) | 94 (35%) |  |
| *High School or above* | 2,823 (83%) | 2,649 (84%) | 174 (65%) |  |
| **PIR (%)** |  |  |  | **<0.001** |
| *Not Poor* | 2,167 (70%) | 2,026 (70%) | 141 (60%) |  |
| *poor* | 946 (30%) | 851 (30%) | 95 (40%) |  |
| **Smoking (%)** |  |  |  | 0.692 |
| *Never* | 2,212 (65%) | 2,034 (65%) | 178 (66%) |  |
| *Former* | 497 (15%) | 457 (15%) | 40 (15%) |  |
| *Current* | 707 (21%) | 657 (21%) | 50 (19%) |  |
| **Physical activity (%)** |  |  |  | 0.171 |
| *Inactive* | 348 (12%) | 318 (12%) | 30 (16%) |  |
| *Active* | 2,456 (88%) | 2,293 (88%) | 163 (84%) |  |
| **Hypertension (%)** |  |  |  | **<0.001** |
| *No* | 2,724 (80%) | 2,532 (80%) | 192 (72%) |  |
| *Yes* | 692 (20%) | 616 (20%) | 76 (28%) |  |
| **Diabetes (%)** |  |  |  | **<0.001** |
| *No* | 3,156 (92%) | 2,927 (93%) | 229 (85%) |  |
| *Yes* | 260 (7.6%) | 221 (7.0%) | 39 (15%) |  |
| **Hyperlipidemia (%)** |  |  |  | **<0.001** |
| *no* | 1,527 (45%) | 1,458 (46%) | 69 (26%) |  |
| *yes* | 1,889 (55%) | 1,690 (54%) | 199 (74%) |  |
| **Neutrophil Percentage (%)** | 56.68 (8.97) | 56.46 (9.04) | 59.28 (7.75) | **<0.001** |
| **Albumin_(g/dL)** | 4.28 (0.35) | 4.29 (0.35) | 4.16 (0.35) | **<0.001** |
| **NPAR (mean (SD))** | 13.33 (2.40) | 13.24 (2.38) | 14.36 (2.29) | **<0.001** |
| **NPAR (%)** |  |  |  | **<0.001** |
| *T1* | 1,144 (33%) | 1,093 (35%) | 51 (19%) |  |
| *T2* | 1,114 (33%) | 1,033 (33%) | 81 (30%) |  |
| *T3* | 1,158 (34%) | 1,022 (32%) | 136 (51%) |  |
| **Phenotypic age (mean (SD))** | 32.63 (9.99) | 32.31 (9.92) | 36.41 (10.05) | **<0.001** |
| **Phenotypic age (%)** |  |  |  | **<0.001** |
| *T1* | 1,071 (31%) | 1,018 (32%) | 53 (20%) |  |
| *T2* | 1,161 (34%) | 1,071 (34%) | 90 (34%) |  |
| *T3* | 1,184 (35%) | 1,059 (34%) | 125 (47%) |  |

Mean (SD) for continuous variables: the P value was calculated by the Students T-test.

Percentages (weighted N, %) for categorical variables: the P value was calculated by the chi-square test.

Abbreviation: NPAR, neutrophil percentage-to-albumin ratio; PIR, Ratio of family income to poverty.


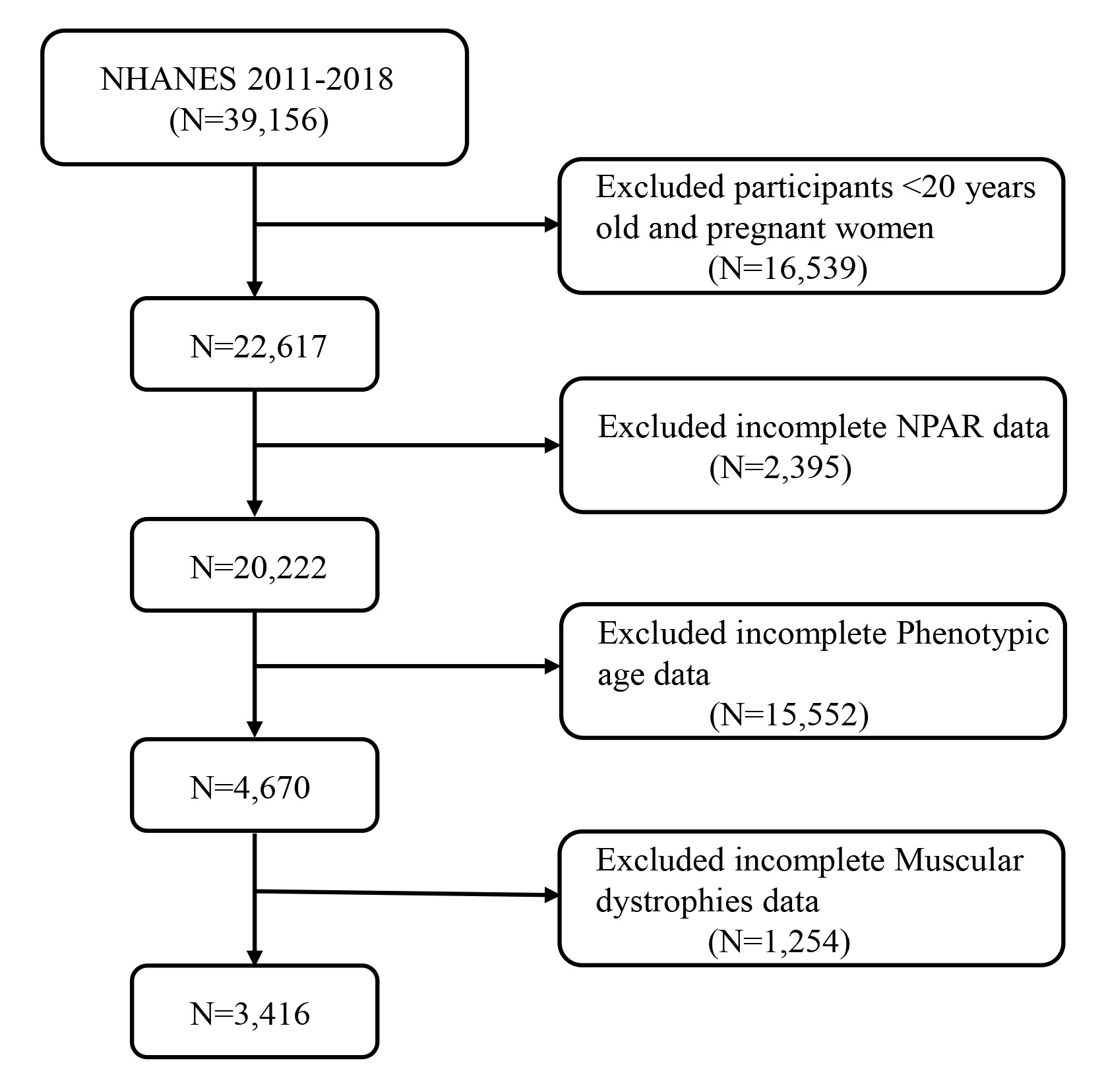


**Figure S1.** A flow diagram of eligible participant selection in the National Health and Nutrition Examination Survey.

Abbreviation: NPAR, neutrophil percentage-to-albumin ratio.
